# Study on plasma metabolomics for HIV/AIDS patients treated by HAART based on LC/MS-MS

**DOI:** 10.3389/fphar.2022.885386

**Published:** 2022-08-29

**Authors:** Donghui Lao, Rong Liu, Jianying Liang

**Affiliations:** ^1^ Department of Pharmacy, Zhongshan Hospital, Fudan University, Shanghai, China; ^2^ Department of Pharmacy, Shanghai Public Health Clinical Center, Fudan University, Shanghai, China; ^3^ School of Pharmacy, Fudan University, Shanghai, China

**Keywords:** metabolomics, HIV, AIDS, biomarker, LC-MS/MS

## Abstract

**Background:** Metabolomics can be applied to the clinical diagnosis and treatment evaluation of acquired immune deficiency syndrome (AIDS). AIDS biomarkers have become a new direction of AIDS research providing clinical guidance for diagnosis.

**Objective:** We sought to apply both untargeted and targeted metabolomic profiling to identify potential biomarkers for AIDS patients.

**Methods:** A liquid chromatography-tandem mass spectrometry (LC-MS/MS) based untargeted metabolomic profiling was performed on plasma samples of patients before and after highly active antiretroviral therapy (HAART) treatment as well as healthy volunteers to identify potential AIDS biomarkers. Targeted quantitative analysis was performed on the potential biomarkers screened from untargeted metabolic profiling for verification.

**Results:** Using the Mass Profiler Professional and the MassHunter, several potential biomarkers have been found by LC-MS/MS in the untargeted metabolomic study. High-resolution MS and MS/MS were used to analyze fragmentation rules of the metabolites, with comparisons of related standards. Several potential biomarkers have been identified, including PS(O-18:0/0:0), sphingosine, PE (21:0/0:0), and 1-Linoleoyl Glycerol. Targeted quantitative analysis showed that sphingosine and 1-Linoleoyl Glycerol might be closely related to HIV/AIDS, which may be the potential biomarkers to the diagnosis.

**Conclusion:** We conducted untargeted metabolomic profiling, which indicates that several metabolites should be considered potential biomarkers for AIDS patients. Further targeted metabolomic research verified that d-Sphingosine and 1-Linoleoyl glycerol as the diagnostic biomarker of AIDS.

## Introduction

Acquired immune deficiency syndrome (AIDS) is a malignant, infectious disease caused by the human immunodeficiency virus (HIV), which can cause great harm and high mortality (Coffin et al., 1986). Although HIV has different clinical manifestations, it has the characteristics of rapid transmission and increased mortality (2–6). HIV mainly infects people through blood transfusion, injection, and sexual contact. It quickly invades the body’s immune system, causing its immune system to be destroyed. Cell immune function is damaged or defective, and the body cannot replicate immunity due to low resistance. As a result, it leads to infection, other immune diseases, and even malignant tumors.

Reverse transcriptase, which can transcribe single-stranded RNA of the virus into double-stranded DNA, has been found to play a crucial role in HIV replication and is, therefore, an essential target of therapeutic drugs. Non-nucleoside reverse transcriptase inhibitors (NNRTIs) can bind to the reverse transcriptase of the HIV and form a stable complex, leading to a change of the active conformation of the enzyme, hence inhibiting its transcription function and blocking the replication of HIV. Highly active antiretroviral therapy (HAART) is a treatment regimen that uses a combination of antiretroviral drugs to combat AIDS, including nucleoside reverse transcriptase inhibitors, non-nucleoside reverse transcriptase inhibitors, and protease inhibitors (Michaels et al., 1998). The HAART therapy can significantly reduce the drug resistance caused by a single drug, effectively inhibit the replication of HIV in the body, and restore the body’s damaged immune system, thereby significantly reducing the morbidity and mortality of AIDS.

The concept of metabolomics was initially derived from metabolic profiling. Nicholson defined *metabolomics* as the multi-parameter quantitively analysis of various metabolic responses in the organism when a living system is subjected to pathological, physiological stimuli, or genetic changes (Nicholson et al., 1999). Metabolomics studies the small molecule metabolites in biological body fluids and tissue systems to analyze physiological and pathological changes or genetic mutations qualitatively and quantitatively physiological and pathological changes or genetic mutations in the body using high-sensitivity, high-throughput, and high-resolution sample analysis techniques and methods. At the same time, impressive mathematical statistics and bioinformatics software were used to classify and predict the changes of metabolites caused by other factors, find potential biomarkers, and make an overall biological assessment of the body’s condition and function.

The current diagnostic indices of AIDS is mainly the viral load in the patient’s body, that is, the amount of HIV RNA in the patient’s plasma or serum, which is an index for the quantitative detection of HIV nucleic acid, and the clinical diagnosis is made through HIV antibody confirmation test (McDaniel et al., 2000). However, there may be uncertainties in using a single diagnostic method. If it can be combined with the corresponding biomarkers in the body to assist diagnosis, the reliability and confidence of the diagnosis can be improved. As the terminal of biological information transmission, the metabolites in the organism can show the final result of the overall function or state of the biological system (Johnson et al., 2016). The study of metabolites can provide a theoretical basis and solutions for elucidating many disease mechanisms. Therefore, metabolomics can be applied to the clinical diagnosis and treatment evaluation of AIDS. The use of AIDS biomarkers can initially understand whether HIV is infected and the degree of infection in the body and provide clinical guidance for AIDS diagnosis. In recent years, it has become a new direction of AIDS research.

## Materials and methods

### Materials

Standards such as d-sphingosine (S7049-5 MG, Lot: MKBV0715V), 1-linoleoyl glycerol (M7640-100 MG, Lot: 091M1689V), and lipid standards (triglyceride mixtures) (17,811-100 MG, Lot: LC14890V) were purchased from Sigma-Aldrich Company (United States). HPLC grade acetonitrile (168,171-4L, Lot: LP50K04) was purchased from J&K Scientific (China). Analytical Reagent grade chloroform (10,006,818-500 ml, Lot: 20,120,105) was purchased from Sinopharm Chemical Reagent Co., Ltd. (China). Deionized water was produced by the Millipore Direct-Q water purification system (Merck Millipore, Guyancort, France).

### Ethics

The Institutional Review Board approved the study protocol of Shanghai Public Health Clinical Center, Fudan University. All procedures performed in studies involving human participants were following the ethical standards of the institutional and national research committee and with the 1964 Helsinki declaration and its later amendments or comparable ethical standards.

### Study population

76 subjects were enrolled in an untargeted metabolomics study, including 38 paired AIDS patients before and after HAART treatment. A total of 10 study subjects were enrolled in a targeted metabolomic study, including five paired AIDS patients before and after HAART treatment. The patients were all required to HIV confirmation test positive, aged between 18 and 60 years old, and had undergone HAART treatment for more than 6 months. 38 cases of healthy volunteers were enrolled in the control group. All healthy volunteers were HIV antibody-negative, aged between 18 and 60, healthy, and had no acute episodes of chronic diseases or severe organ diseases, and females were not pregnant or breastfeeding. All AIDS patients and healthy volunteers were enrolled from Shanghai Public Health Clinical Center, Fudan University. All AIDS patients and healthy controls were male and of Han nationality. No other demographic information was collected due to patient privacy concerns.

### Sample collection

Blood samples were collected from 38 control subjects and 38 paired AIDS patients before and after HAART treatment. All the samples were transferred into heparinized tubes and stored in the refrigerator. The test solution was processed as follows: thaw the plasma sample at 4°C, accurately draw 150 μl aliquot the plasma sample into an EP tube, add 450 μl of cold acetonitrile (stored at 4°C for 24 h) for protein precipitation, vortex for 1 min, stand at 4°C for 10 min, centrifuge at 12,000 r/min for 10 min, and transfer the supernatant to an EP tube, blow dry with nitrogen at a constant temperature of 40°C, and finally add 100 μl of acetonitrile: water (3:1, v/v), use ultrasonic to disperse the solution. An aliquot of 10 μl of test solution was injected for UHPLC/MS analysis.

### UHPLC-QTOF/MS

Plasma samples were analyzed using Agilent 1260 high-resolution liquid chromatography and 6,520 Accurate-Mass QTOF mass spectrometry (Agilent Corporation, Santa Clara, CA, United States). Chromatographic separation of metabolites was performed using a Waters Acquity UPLC HSS T3 Column (2.1 mm × 100 mm, 1.8 μm particle size) with a gradient of 100% water with 0.1% formic acid (Solvent A) to 100% acetonitrile with 0.1% formic acid (Solvent B). The solvent flow rate was 300 μl/min, the column temperature was 35 °C, and the sample temperature was 4°C. The gradient elution program is shown in Table 1.

**TABLE 1:** Gradient elution program.

Time (min)	Solvent A (%)	Solvent B (%)	Flow (μL/min)
0	99	1	300
1	99	1	300
5	60	40	300
8	50	50	300
10	35	65	300
16	24	76	300
20	0	100	300
25	0	100	300
27	99	1	300

Mass spectrometric detection was carried out in both positive and negative ion scan mode. Instrument parameters were set: capillary voltage 3500 V, gas temperature 350°C, and collision energy 20 V. A first-level full scan mode was employed in the 50–1000 m/z mass range. High-purity nitrogen is used in all gas circuits.

MassHunter mass spectrometer workstation, Mass Profiler Professional (MPP) data processing software, DA Reprocessor software (Agilent Technologies, Germany) are used for data acquisition and processing. XCMS-Online (https://xcmsonline.scripps.edu) is used to identify the accurate mass number of every peak.

### Untargeted metabolomic profiling

Import the above-obtained data into MPP for multivariate statistical analysis, and use unsupervised analysis to obtain Principal Component Analysis (PCA) diagrams, 3D PCA diagrams, and Correlation-Covariance (C-C) diagrams more intuitively distinguish different metabolites among different data groups. Then use Volcano Plot and hierarchical cluster analysis to confirm the discrimination results. Finally, the compounds with significant differences between the two groups were screened out based on the analysis results. Then the *t*-test was performed on them, and the data of the compounds with *p* < 0.05 and the Fold Change between the two groups were greater than 2. By comparing the changes of the corresponding metabolites between the two groups, statistical analysis clarifies its changing trend, such as increase or decrease, up-regulation, or down-regulation. Finally, search and compare the human metabolome database METLIN, HMDB and NIST according to its accurate mass to find potential biomarkers.

### Targeted metabolomic profiling

A targeted quantitative analysis was performed to analyze four potential biomarkers screened from the untargeted metabolomic study. Using the two related standards of discriminating metabolites, samples from five AIDS patients and five healthy volunteers have been quantitatively analyzed.

## Results

### Analytical method validation

*Blank solvent:* Take the blank solvent, acetonitrile: water (3:1, v/v, to prepare the sample solution and inject the sample according to the conditions in the gradient elution table.

*Precision test:* repeat the determination of the sample solution for 5 times, verify the precision of the instrument according to the retention time and peak area of each chromatographic peak in the total ion flow diagram, and the RSD value is less than 20%.

*Repeatability test:* take the same plasma sample and prepare five samples in parallel for determination. According to the retention time and peak area of each chromatographic peak in the total ion flow diagram, verify the repeatability of the method, and the RSD value is less than 20%

*Stability test:* determine the test solution at 0, 2, 4, 8, 12, and 24 h, respectively. Take the retention time and peak area of each color spectrum peak of the total ion flow diagram as indicators to verify the stability of the sample. The RSD values are less than 20%.

The applied method was validated before analyzing the plasma samples, including the precision, repeatability, and within-day stability of sample preparation.

### Separation

**FIGURE 1:**
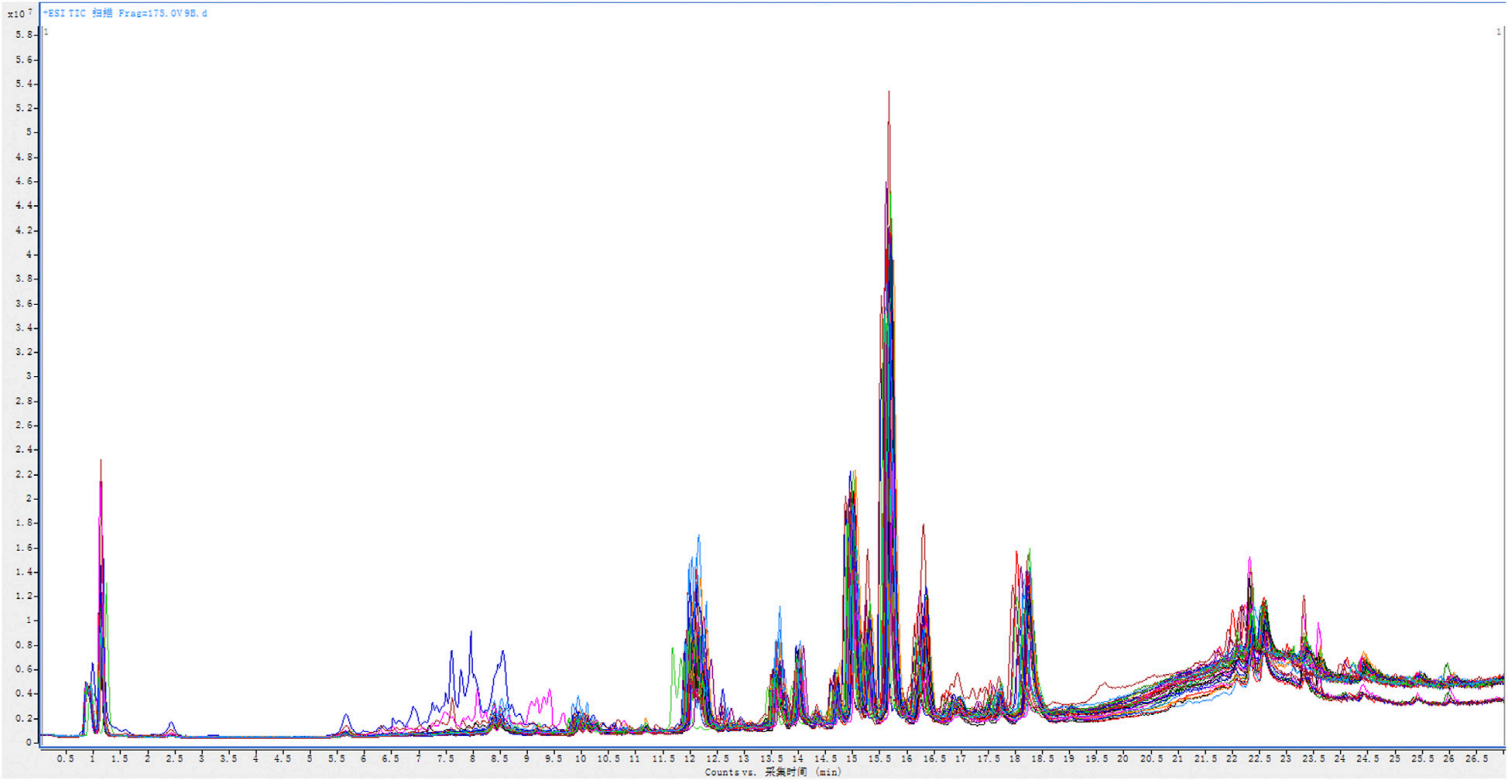
The superimposed total ion chromatogram (TIC) of the plasma samples from 38 healthy volunteers and 38 AIDS patients before and after HAART treatment.

The established high-resolution LC-MS/MS analysis conditions were used to retrospectively determine the plasma samples of 38 healthy volunteers and 38 AIDS patients before and after HAART treatment. Under positive ion detection mode, the plasma samples of the three groups are all well separated. The superimposed total ion chromatogram (TIC) of each group is shown in Figure 1.

### Untargeted metabolomic profiling

#### Volunteers vs patients before HAART treatment

**FIGURE 2:**
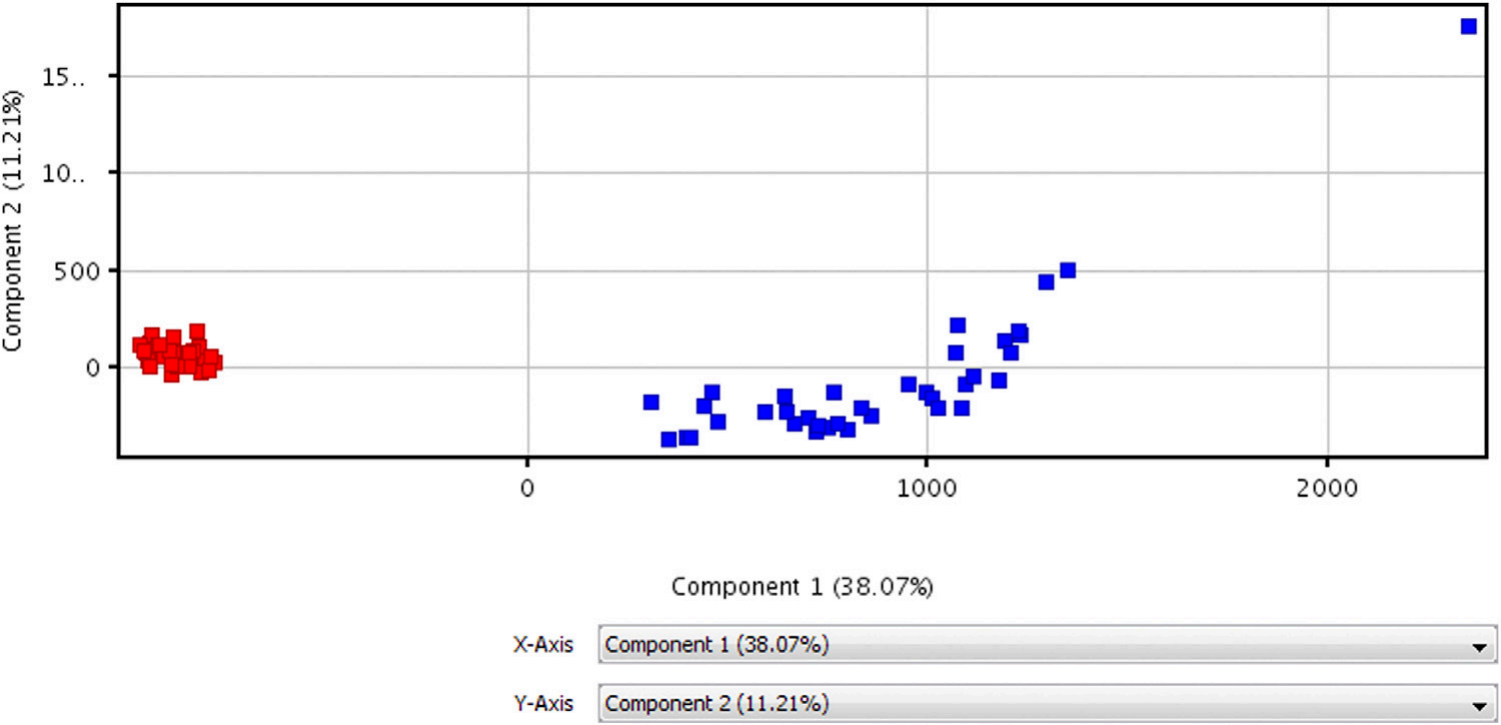
PCA diagram for untargeted metabolomic profiling of healthy volunteers vs patients before HAART treatment.

**FIGURE 3:**
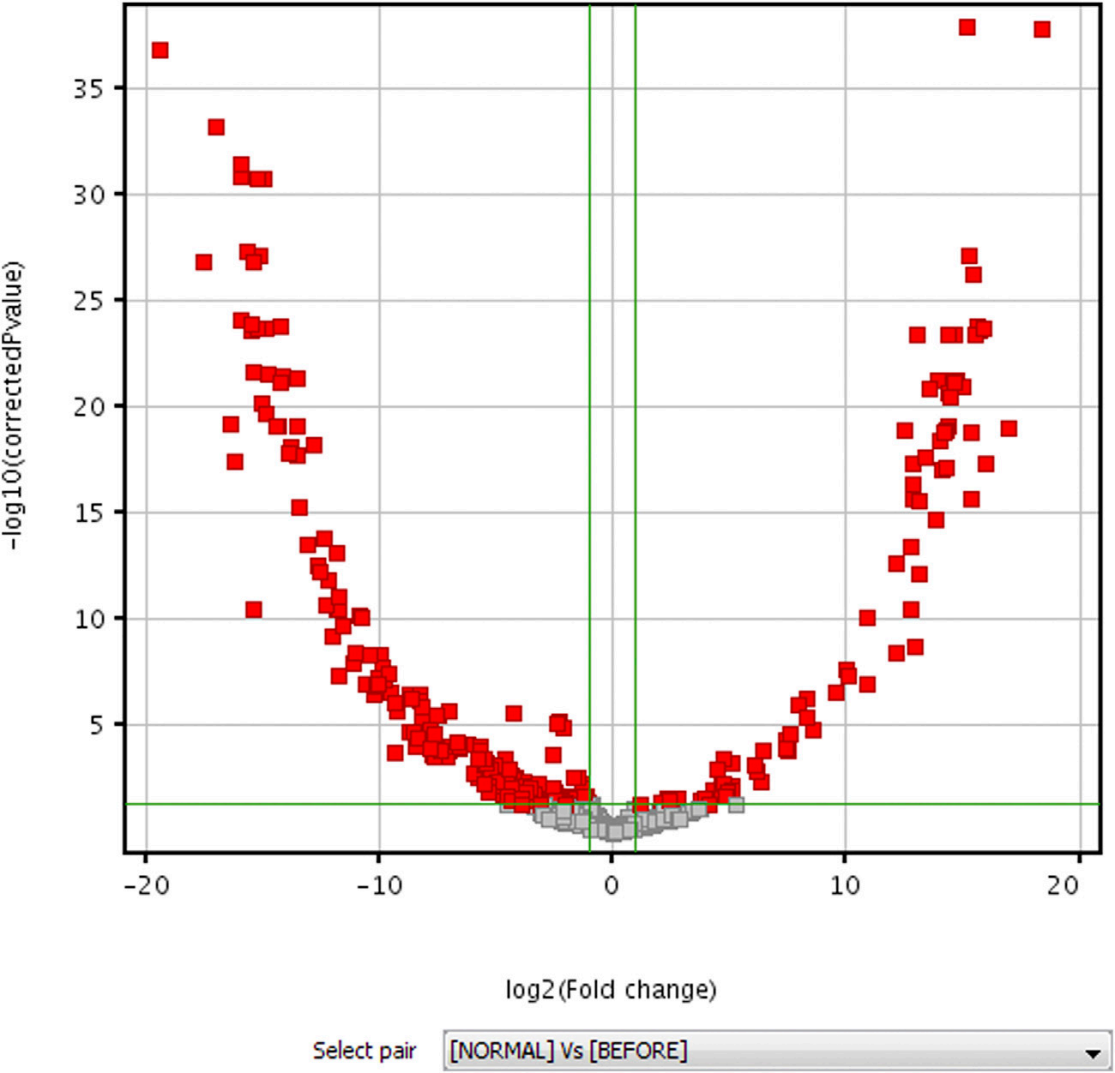
Volcano plot diagram for untargeted metabolomic profiling of healthy volunteers vs patients before HAART treatment.

**FIGURE 4:**
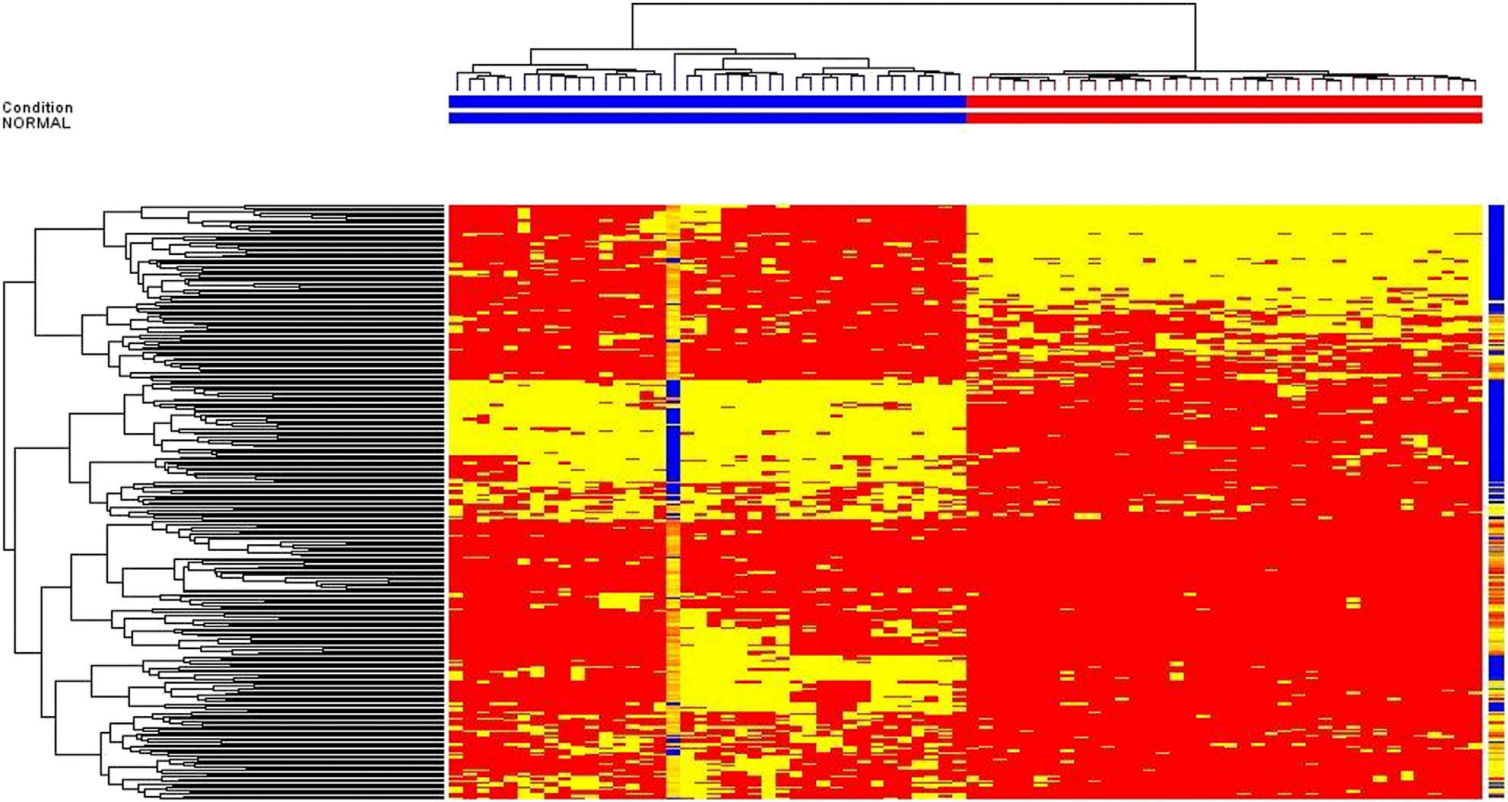
Hierarchical cluster analysis for untargeted metabolomic profiling of healthy volunteers vs patients before HAART treatment.

The data of volunteers and patients before HAART treatment are statistically analyzed after quality control, using MPP data processing software. Different possible metabolites were screened out according to PCA diagram (see Figure 2 for details), volcano plot diagram (see Figure 3 for detials), hierarchical cluster analysis (see Figure 4 for details), and *t*-test.

By searching the MPP software and the human metabolome database HMDB, NIST, and METLIN, the relevant information of the substance was determined according to the precise mass, and 15 different compounds were initially compared. See Table 2 For details.

**TABLE 2:** Differential compounds of healthy volunteers and patients before HAART treatment.

No.	Retention time	m/z	Compound	*p* value
1	0.9555	129.0436	Pyrroline hydroxycarboxylic acid	5.94 × 10^−26^
2	1.1436	341.1505	His-Trp	3.36 × 10^−23^
3	8.8855	332.1384	16-bromo-9E-hexadecenoic acid	8.56 × 10^−41^
4	11.4311	511.3293	PS(O-18:0/0:0)	9.97 × 10^−26^
5	13.5238	299.2909	Sphingosine	0.00
6	17.6605	183.0666	Indole-3-ethanol	1.87 × 10^−25^
7	17.3406	302.2260	Methenolone	4.65 × 10^−19^
8	17.6646	523.3666	PE (21:0/0:0)	3.06 × 10^−23^
9	20.8519	354.2788	1-Linoleoyl Glycerol	7.06 × 10^−19^
10	20.4537	561.4175	CerP (d18:1/12:0)	1.39 × 10^−28^
11	0.7943	83.9531	Dichloromethane	0.00
12	1.0990	292.0922	EDTA	3.50 × 10^−29^
13	7.0402	458.2741	Arg-Arg-Gln	6.33 × 10^−21^
14	14.9371	329.3312	*N,N*-dimethyl-Safingol	1.82 × 10^−29^
15	4.6323	961.0683	TG (15:0/20:0/24:0)	5.20 × 10^−21^

Only His-Trp, PS (O-18:0/0:0), Sphingosine, PE (21:0/0:0), 1-Linoleoyl Glycerol, CerP (d18:1/12:0), Arg-Arg-Gln, and triglycerides are endogenous metabolites among the 15 different compounds obtained. Therefore, these eight differential compounds are possible potential biomarkers. The other differential compounds are exogenous and should not be considered.

#### Patients before HAART treatment vs patient after HAART treatment

Search the eight potential biomarkers obtained above by comparing the fold changes of the corresponding potential biomarkers between the patients before and after HAART treatment. The fold change is clarified. Based on this, it can be found which metabolites in AIDS patients will be affected by HAART treatment. The results are shown in Table 3.

**TABLE 3:** The fold changes of the potential biomarkers between the patients before and after HAART treatment.

Compound	Retion time	m/z	Fold change	Regulation	P value
His-Trp	1.1431	341.1505	1.3062	Down	9.25 × 10^−05^
PS(O-18:0/0:0)	11.4308	511.3293	5.2738	Up	4.65 × 10^−04^
Sphingosine	13.5204	299.2909	2.6306	Up	1.09 × 10^−05^
PE (21:0/0:0)	17.6563	523.3666	3.3002	Up	8.21 × 10^−04^
1-Linoleoyl Glycerol	20.8657	354.2788	5.1069	Down	4.19 × 10^−04^
CerP (d18:1/12:0)	20.4519	561.4175	1.3402	Up	1.15 × 10^−08^
Arg-Arg-Gln	7.0420	458.2741	1.7076	Down	1.71 × 10^−04^
TG (15:0/20:0/24:0)	4.6323	961.0683	1.3365	Down	8.82 × 10^−10^

According to the results above, PS (O-18:0/0:0), sphingosine, PE (21:0/0:0), and TG (15:0/20:0/24:0) were up-regulated to a corresponding extent after HAART treatment. However, His-Trp and 1-linoleic acid glyceride were lowered to a certain extent after HAART treatment. Compared with the healthy control group, these six different metabolites in patients after HAART treatment tend to become normal and are likely to be biomarkers. In addition, CerP (d18:1/12:0) and Arg-Arg-Gln should be excluded from biomarkers, as the fold change in the patient group after HAART treatment is further away from the healthy control group compared with the patient group before HAART treatment.

#### Targeted metabolomic profiling

We analyzed the plasma samples of five healthy volunteers and five AIDS patients before and after HAART treatment. The plasma concentrations were determined in humans by measuring the peak areas of d-Sphingosine and 1-Linoleoyl glycerol in the actual plasma samples. By comparing the changes in the concentrations of the different metabolites between the three groups, the diagnostic biomarkers of AIDS were verified. The results are shown in Table 4.

**TABLE 4:** The fold changes of the potential biomarkers between the patients before and after HAART treatment.

Metabolite	Healthy volunteers (n = 5)		Patients before HAART treatment (n = 5)		Patients after HAART treatment (n = 5)	
	Area	Concentration (ng/mL)	Area	Concentration (ng/mL)	Area	Concentration (ng/mL)
d-Sphingosine	54,536.46	60.63	1973.43	-	6,842.89	4.23
	28,520.89	29.87	2,816.11	-	18,245.81	17.72
	97,945.82	111.97	3,474.94	-	12,888.78	11.38
	39,440.69	42.78	-	-	22,038.53	22.20
	67,985.21	76.54	1123.45	-	37,928.51	40.99
1-Linoleoyl glycerol	14,404.63	229.36	987,541.88	22,934.38	215,547.23	4,922.37
	64,982.55	1409.43	1,675,423.20	38,983.88	121,456.10	2,727.06
	33,517.33	675.29	478,965.52	11,068.39	46,527.83	978.85
	27,514.17	535.23	3,316,574.12	77,274.84	98,997.15	2,203.05
	9,836.05	122.77	1,088,452.41	25,288.80	306,541.84	7,045.44

The results showed that the plasma levels of sphingosine in AIDS patients before HAART treatment were significantly lower than those in healthy volunteers. After HAART treatment, the plasma levels of sphingosine in patients increased to a certain extent and tended to return to normal levels. The concentration of 1-linoleoyl acid glycerol in the plasma of AIDS patients before HAART treatment was significantly higher than that of healthy volunteers. After HAART treatment, the plasma concentration of 1-linoleic acid glyceride was down-regulated tended to return to normal levels.

## Discussion

Mass Profiler Professional, Agilent’s latest metabolomics statistical analysis software, has principal component analysis, cluster analysis, partial least squares discriminant analysis, *t*-test, and other multivariate statistical functions. MPP software is a more convenient and consistent tool for metabolomics research than traditional approaches such as SIMCA, Cluster, and R (Barupal et al., 2018; Caesar et al., 2018; Wallace et al., 2020).

With the increasing improvement of detection and analysis methods, metabolomics has made considerable progress in AIDS research in recent years, becoming a research hotspot in the clinical diagnosis of AIDS worldwide. Currently, limited literature on metabolomics research on AIDS plasma in the Chinese population is reported. Therefore, our research used the detection of endogenous small molecule metabolites in plasma of AIDS patients to analyze the overall metabolite response changes in the body caused by HIV, assisting disease diagnosis by discovering AIDS-related biomarkers. Wikoff et al. used LC-MS technology to analyze the cerebrospinal fluid of monkeys infected with simian immunodeficiency virus (SIV). They found three potential biomarkers: stearic acid, octadecenoic acid, and arachidonic acid (Wikoff et al., 2008). Hollenbaugh et al. used LC-MS/MS to simultaneously perform metabolomics analysis on long-term HIV-infected cells and CD4^+^ T lymphocytes. The results showed that CD4^+^ T lymphocytes have greater energy uptake than long-term HIV-infected cells (Hollenbaugh et al., 2011). We established a targeted quantitative analysis method and an untargeted metabolomics research method for plasma samples of AIDS patients by rapid resolution liquid chromatography-tandem mass spectrometry (RRLC-MS/MS) technology. We finally screened out six different metabolites closely related to the diagnosis of AIDS and confirmed the two of them, d-Sphingosine and 1-Linoleoyl glycerol, with high-resolution MS/MS spectra through standard products. These metabolites are very likely to be a potential biomarker for AIDS diagnosis.

The healthy control group, patients before the HAART treatment group, and patients after the HAART treatment group were compared pairwisely. After multivariate statistical analysis and screening with the human metabolome database, six possible potential biomarkers were finally screened, such as His-Trp, PS(O-18:0/0:0), sphingosine, PE (21:0/0:0), 1-Linoleoyl glycerol, TG (15:0/20:0/24:0). These potential biomarkers may be related to the pathophysiology of AIDS and play an essential role in the energy metabolism of sugar, fat, and protein, cell damage and repair, biosynthesis, and inflammation. Their changes will have multiple effects on body-related metabolism.

PS (O-18:0/0:0) is synthesized by the human body using serine and exists in the human biofilm. It is one of the essential components of cell membrane phospholipids, accounting for 10–20% of all phospholipids in the brain. It plays a critical regulatory role in many cell metabolic processes and can activate Na^+^/K^+^ - ATPase, protein kinase C, and tyrosine hydroxylase on the cell membrane (Vance and Steenbergen, 2005). In addition, phosphatidylserine can undergo a methylation reaction to produce phosphatidylcholine, which can be used as a precursor for synthesizing acetylcholine, thus participating in the regulation of cell membrane fluidity (Chua et al., 2019). Phosphatidylserine can modulate the fluidity of the cell membrane and influence the infection ability of HIV. Therefore, its content adjustment *in vivo* has a specific reference value for diagnosing and preventing AIDS (Soares et al., 2008).

Sphingosine is one of the metabolites of sphingolipids, and it is the structural component of the cell membrane. At the same time, it also has the biological activity of signal transmission. It can be used as the first and second messenger to regulate the life activities of cells, such as cell survival, proliferation, migration, neovascularization, etc. (Payne et al., 2002). Because HIV binds to CD4 + receptor amino acid residues on the human target cell membrane through glycoprotein, it attaches to the surface of target cells. The metabolic changes of sphingosine change the components of the cell membrane indirectly affect the infection ability of HIV.

PE (21:0/0:0) is a kind of phospholipid. It is produced by the reaction of 1,2-diglyceride and CDP ethanolamine in the human body. Its content is second only to lecithin in the phospholipids existing in the biological world. In different species, the phosphatidylethanolamine composed in fatty acids is also different. There are more saturated fatty acids in microorganisms and egg yolk than in animal tissues. Phosphatidylethanolamine can produce lysophosphatidic ethanolamine (LPE) under the action of phospholipase A *in vivo*. It has been reported that LPE has significant medical research value in HIV resistance. Therefore, the adjustment of its precursor PE (21:0/0:0) *in vivo* can affect the infection ability of HIV. It can be used as a biomarker for AIDS diagnosis and evaluation (Chai et al., 2019).

1-Linoleoyl glycerol is produced by the reaction of linoleic acid and glycerol, and its content in the human body is low. Linoleic acid is an essential fatty acid that can reduce blood cholesterol and prevent atherosclerosis. Glycerol is the final product of triglycerides in fat ingested by the human body after metabolism and decomposition *in vivo*, stored in fat cells. Studies have shown that untreated patients with severe HIV infection will decrease triglyceride levels related to lipid metabolism disorders (Souza et al., 2013). Therefore, the state of 1-linoleic acid glyceride *in vivo* can indirectly evaluate triglyceride levels *in vivo* and be used as one of the indicators of HIV diagnosis.

TG (15:0/20:0/24:0) is produced by the reaction of glycerol and long-chain fatty acids. They are the most abundant lipids in the body and supply energy for most tissues and organs. Triglycerides can be synthesized in the human liver, adipose tissue, intestinal mucosal epithelial cells and stored in adipose tissue. The metabolic pathway of TG *in vivo* is that the liver absorbs chylomicrons (CM) hydrolysates and residues through receptor-mediated, and its derivatives synthesize very-low-density lipoprotein (VLDL) with some new components (Kwiterovich, 2000). The study found that the levels of triglycerides, high-density lipoprotein cholesterol, and low-density lipoprotein cholesterol in untreated patients with severe HIV infection usually decreased simultaneously (Rose et al., 2008). As the metabolism of triglycerides in the body was enhanced after HIV infection, its level is significantly lower than in the healthy body. Therefore, triglyceride is an objective and vital biomarker for HIV prevention and diagnosis.

The number of differential metabolites obtained in the untargeted metabolomics study is challenging. Studies utilizing metabolomics data are risking unreliable reporting results if peaks cannot be detected for low-sensitivity or low-quality integrations from untargeted LC-MS/MS were not removed (Chetnik et al., 2020). Therefore, it is necessary to optimize the conditions of data quality control to make the metabolite change information more comprehensive and complete.

Disciplines such as metabolomics, genomics, and proteomics are not isolated research fields. Omics research is often interrelated, and it is necessary to coordinate in the research process (Christians et al., 2016; Schrimpe-Rutledge et al., 2016; Telenti, 2018). The results of AIDS metabolomics research can be further combined with genomics, proteomics, and other research to deeply explore the body’s overall condition and grasp the changes before and after the onset of AIDS patients from the level of systematic biology.

## Conclusion

We conducted untargeted metabolomic profiling, which indicates that PS(O-18:0/0:0), sphingosine, PE (21:0/0:0), and TG (15:0/20:0/24:0), His-Trp, and 1-Linoleic acid glyceride should be considered potential biomarkers for AIDS patients. Further targeted metabolomic research verified that d-Sphingosine and 1-Linoleoyl glycerol as the diagnostic biomarker of AIDS.

## Data Availability

The raw data supporting the conclusion of this article will be made available by the authors, without undue reservation.
